# Baloxavir Exhibits Antibacterial Activity Against *Staphylococcus aureus* by Inhibiting De Novo Purine Biosynthesis

**DOI:** 10.3390/ijms27093880

**Published:** 2026-04-27

**Authors:** Xue Li, Yan Yang, Penghe Wang, Tongying Nie, Xinxin Hu, Xuefu You, Xiukun Wang, Congran Li

**Affiliations:** 1 Beijing Key Laboratory of Technology and Application for Anti-Infective New Drugs Research and Development, Institute of Medicinal Biotechnology, Chinese Academy of Medical Sciences & Peking Union Medical College, Beijing 100050, China; xueli@imb.pumc.edu.cn (X.L.); wangpenghe@imb.pumc.edu.cn (P.W.);; 2 Division for Medicinal Microorganisms-Related Strains, CAMS Collection Center of Pathogenic Microorganisms, Beijing 100050, China; 3 Hebei Key Laboratory of Pathogenic Mechanisms and Diagnosis & Treatment Technologies for Lung Microbiome, Zhangjiakou 075000, China; 4 Medical Research Center, The Affiliated Hospital of Southwest Jiaotong University, The Third People’s Hospital of Chengdu, Chengdu 610031, China; yangyan_y@163.com

**Keywords:** baloxavir, *Staphylococcus aureus*, antibacterial, intracellular, purine biosynthesis

## Abstract

*Staphylococcus aureus* remains a leading cause of morbidity and mortality worldwide, with persistent and relapsing infections posing a major global health threat. Here, we report that baloxavir, an FDA-approved *influenza* antiviral, exhibits antibacterial activity against *S. aureus*. Baloxavir demonstrated potent activity against both MSSA and MRSA clinical isolates with MICs of 2–4 μg/mL and exhibited concentration-dependent antibacterial activity in time-kill assays. Notably, baloxavir effectively eliminated intracellular *S. aureus* in both A549 alveolar epithelial cells and RAW264.7 macrophages at 10 μg/mL and achieved complete eradication in A549 cells at 50 μg/mL. In vivo, baloxavir (20–40 mg/kg) significantly improved survival in MRSA-infected mice from 12.5% to 75–87.5%. Transcriptomic analysis revealed significant downregulation of purine de novo biosynthesis genes, including *purF* and *purK*, which was validated by RT-qPCR (r = 0.862, *p* = 0.027). This study demonstrates for the first time that baloxavir possesses significant antibacterial activity against *S. aureus* including MRSA, positioning it as a promising repurposed candidate for treating persistent intracellular infections and post-viral superinfections.

## 1. Introduction

*Staphylococcus aureus* (*S. aureus*), including methicillin-resistant *S. aureus* (MRSA), remains one of the most clinically significant bacterial pathogens globally. According to the Global Burden of Disease 2021 Antimicrobial Resistance Collaborators, *S. aureus* was the pathogen with the greatest increase in attributable mortality over the past three decades [1]. Beyond its role as a primary pathogen, *S. aureus* represents a critical secondary invader following viral respiratory infections, accounting for >90% of deaths in the 1918 *influenza* pandemic [2] and 20–30% of co-infections during the 2009 H1N1 pandemic [3].

*S. aureus* is clinically notorious for causing persistent and recurrent infections which are associated with extremely high relapse rates [4,5]. Although traditionally considered an extracellular pathogen, *S. aureus* can invade and survive within various host cell types, including epithelial cells, endothelial cells, and macrophages [6,7,8]. Within these intracellular niches, bacteria may adopt metabolically inactive phenotypes, including small colony variants [9], which exhibit enhanced persistence and contribute to recurrent infections [8]. Importantly, many anti-staphylococcal agents, such as vancomycin, exhibit limited intracellular penetration, leaving this reservoir largely inaccessible to treatment. Consequently, there is an urgent need for new therapeutic agents against *S. aureus*, especially those capable of eliminating intracellular *S. aureus*.

The development of novel antibiotics has lagged far behind the emergence of resistance [10]. The last truly novel antibiotic class, the lipopeptides (daptomycin), was discovered in 1987, creating a “discovery void” spanning more than three decades [11]. Traditional antibiotic development requires 10–15 years and costs approximately $1–2 billion per compound, with low success rates and limited return on investment driving multiple pharmaceutical companies to exit the field [12]. Drug repurposing offers an attractive alternative strategy, potentially reducing development timelines and costs by utilizing existing safety and pharmacokinetic data [13].

Baloxavir marboxil (BM) is a first-in-class *influenza* antiviral approved by the FDA in October 2018 [14,15]. BM is a prodrug that is rapidly hydrolyzed to its active form, baloxavir acid, following oral administration [16]. The drug inhibits cap-dependent endonuclease in the *influenza* polymerase acidic protein, blocking viral mRNA transcription [17]. Baloxavir exhibits favorable pharmacokinetic properties, including a long elimination half-life of 79–94 h enabling single-dose efficacy, a large volume of distribution (1180 L, ~17 L/kg), and an excellent safety profile in clinical trials [18,19]. To date, no antibacterial activity has been reported for baloxavir.

In the present study, we comprehensively evaluated the antibacterial activity of baloxavir against *S. aureus*, including both methicillin-susceptible and -resistant strains. We assessed in vitro activity, intracellular antibacterial effects, in vivo efficacy, and explored the underlying mechanism of action through transcriptomic analysis. Our findings reveal a novel antibacterial application for this approved antiviral drug, with potential implications for treating staphylococcal infections, particularly in the context of viral–bacterial co-infections.

## 2. Results

### 2.1. Baloxavir Exhibits Selective Antibacterial Activity Against Gram-Positive Bacteria

**Figure 1:**
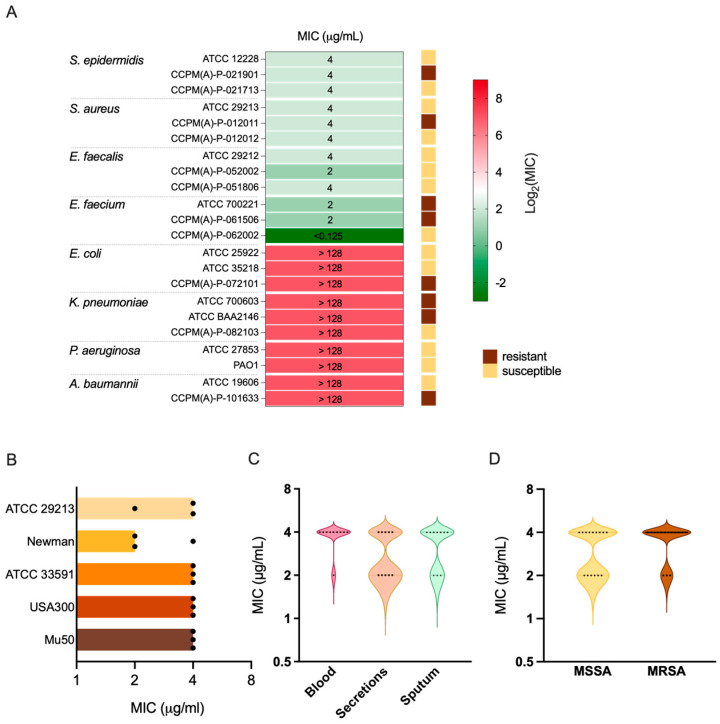
Antibacterial spectrum and activity of baloxavir against *S. aureus*. (**A**) Heatmap showing MIC values of baloxavir against Gram-positive and Gram-negative bacterial species. Color scale indicates log_2_(MIC) values; side annotations indicate methicillin/vancomycin/ESBL/carbapenem resistance phenotype. (**B**) MIC values of baloxavir against *S. aureus* reference strains. Bar plots show individual MIC determinations from three independent experiments. (**C**) MIC distribution of baloxavir against 30 clinical *S. aureus* isolates grouped by specimen source (*n* = 10 per group). (**D**) MIC distribution comparing MSSA (*n* = 15) and MRSA (*n* = 15) clinical isolates.

We first evaluated the antibacterial spectrum of baloxavir against a panel of representative Gram-positive and Gram-negative bacteria, including both methicillin-susceptible and -resistant strains (Table S1, Figure 1A). Baloxavir demonstrated selective activity against Gram-positive bacteria. Against *staphylococci*, including both *S. epidermidis* and *S. aureus*, the MIC was consistently 4 μg/mL regardless of methicillin resistance status. Notably, baloxavir also exhibited activity against *enterococci*, with MICs ranging from <0.125 to 4 μg/mL, including vancomycin-resistant *E. faecium* (VRE). In contrast, no antibacterial activity was observed against Gram-negative bacteria tested (MIC > 128 μg/mL), including *Escherichia coli*, *Pseudomonas aeruginosa*, *Klebsiella pneumoniae*, and *Acinetobacter baumannii*.

### 2.2. Baloxavir Demonstrates Consistent Activity Against S. aureus Strains

Given the clinical importance of *S. aureus*, we evaluated the activity of baloxavir against a broader collection of reference strains and clinical isolates (Figure 1B–D). For the methicillin-susceptible *S. aureus* (MSSA) Newman and ATCC 29213, MIC values were 2 and 4 μg/mL, respectively. Similarly, the MICs against MRSA strains ATCC 33591 and LAC (USA300) were 4 μg/mL. Notably, baloxavir retained activity against the vancomycin-intermediate *S. aureus* (VISA) strain Mu50, with an MIC of 4 μg/mL.

We further tested the antibacterial activity of baloxavir against 30 clinical *S. aureus* isolates collected from various specimen types, including blood (*n* = 10), sputum (*n* = 10), and secretions (*n* = 10) (Table S2). The isolates comprised both MSSA (*n* = 15) and MRSA (*n* = 15) strains. MICs ranged from 2 to 4 μg/mL across all tested clinical isolates, with MIC_50_ and MIC_90_ values both at 4 μg/mL. The MIC distribution was consistent regardless of specimen source (Figure 1C) or methicillin resistance phenotype (Figure 1D), demonstrating uniform susceptibility across diverse *S. aureus* strains.

### 2.3. Baloxavir Exhibits Concentration-Dependent Antibacterial Activity

**Figure 2:**
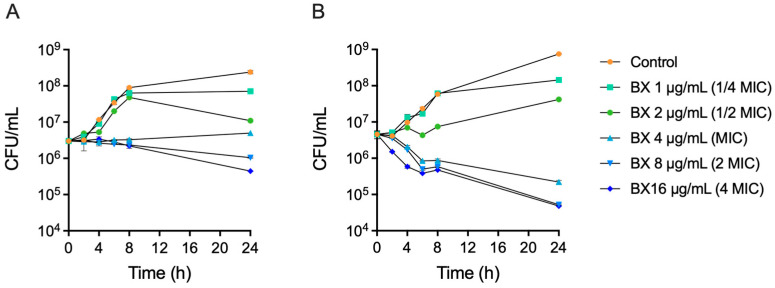
Time-kill assays of baloxavir against *S. aureus*. Time-kill curves of Baloxavir (BX) against (**A**) MRSA reference strain ATCC 33591 and (**B**) clinical MRSA isolate CCPM(A)-P-0116173. Data represent mean ± SEM.

Time-kill assays were performed against MRSA ATCC 33591 and a clinical MRSA isolate CCPM(A)-P-0116173 to characterize the dynamic antibacterial properties of baloxavir (Figure 2). Baloxavir exhibited concentration-dependent antibacterial activity against both strains. At sub-MIC concentrations (1/4× and 1/2× MIC), bacterial growth was delayed but not inhibited. At MIC and higher concentrations, bacterial growth was suppressed. At 4× MIC, baloxavir resulted in a 1 to 2 log10 CFU/mL reduction in bacterial counts by 24 h, indicating a bacteriostatic rather than bactericidal effect under the conditions tested.

### 2.4. Baloxavir Effectively Eliminates Intracellular S. aureus

**Figure 3:**
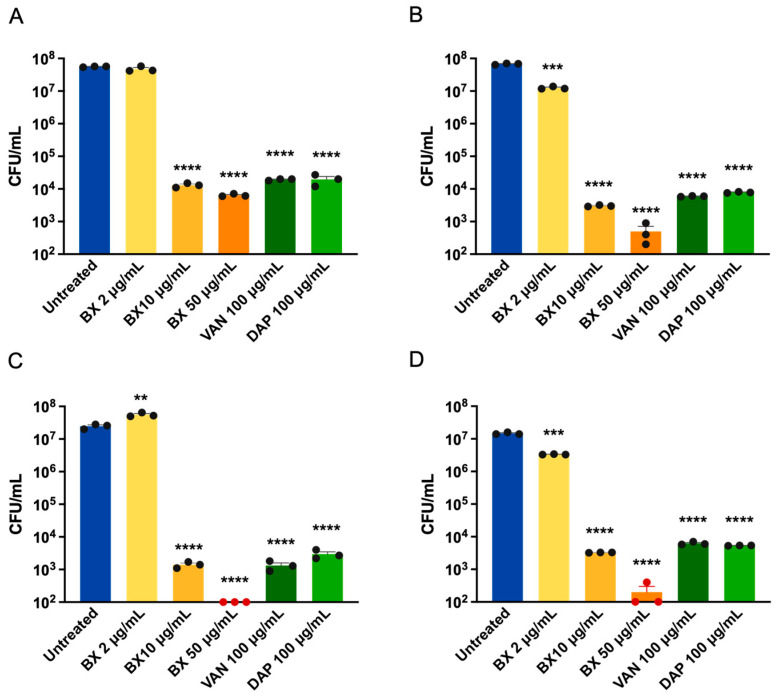
Baloxavir eliminates intracellular *S. aureus* in macrophages and lung epithelial cells. Intracellular antibacterial activity of baloxavir in (**A**,**B**) RAW264.7 murine macrophages and (**C**,**D**) A549 human alveolar epithelial cells infected with *S. aureus* strains Newman (**A**,**C**) or LAC (**B**,**D**). Intracellular bacteria were quantified by cell lysis and CFU enumeration. Bars represent mean ± SEM from three independent experiments. (** *p* < 0.01, *** *p* < 0.001, **** *p* < 0.0001, one-way ANOVA with Dunnett’s multiple comparisons test). BX: baloxavir; VAN: vancomycin; DAP: daptomycin.

The ability of antibiotics to penetrate host cells and kill intracellular bacteria is critical for treating chronic *S. aureus* infections. We assessed the intracellular antibacterial activity of baloxavir in murine RAW264.7 macrophages and human A549 alveolar epithelial cells infected with S. aureus Newman (MSSA) and LAC (MRSA) (Figure 3).

Baloxavir demonstrated concentration-dependent elimination of intracellular bacteria in both cell types. At a sub-inhibitory concentration of 2 μg/mL (1/2 MIC), baloxavir showed negligible intracellular clearance against strain Newman in either cell line, whereas a slight reduction in bacterial load was observed for strain LAC. In contrast, treatment with 10 μg/mL baloxavir resulted in significant bacterial reductions for both strains across both cell types. In RAW264.7 macrophages, treatment with 10 μg/mL baloxavir reduced intracellular Newman by 3.638 ± 0.07 log_10_CFU compared to untreated controls (Figure 3A), and LAC by 4.35 ± 0.11 log_10_CFU (Figure 3B). Similar results were observed in A549 epithelial cells, with reductions of 4.25 ± 0.08 and 3.65 ± 0.11 log_10_CFU for Newman and LAC (Figure 3C,D), respectively. Notably, baloxavir at 10 μg/mL achieved comparable intracellular killing to vancomycin and daptomycin at 100 μg/mL (10-fold higher concentration). Vancomycin and daptomycin were used at 100 μg/mL because preliminary dose-ranging experiments showed that reducing the concentration to 20 μg/mL did not significantly alter intracellular killing efficacy, suggesting that the limited intracellular activity of these antibiotics is due to poor cellular penetration rather than insufficient extracellular drug exposure.

At 50 μg/mL, baloxavir achieved near-complete elimination of intracellular bacteria in macrophages, with reductions of 3.94 ± 0.16 and 5.21 ± 0.11 log_10_CFU for strains Newman and LAC (Figure 3A,B), respectively. In A549 epithelial cells, treatment at this concentration resulted in complete eradication (below the limit of detection) for strain Newman (Figure 3C) and near-complete clearance for strain LAC (Figure 3D). Notably, the intracellular efficacy of baloxavir was superior in A549 epithelial cells compared to that in macrophages.

### 2.5. Baloxavir Marboxil Protects Mice Against Lethal MRSA Infection

**Figure 4:**
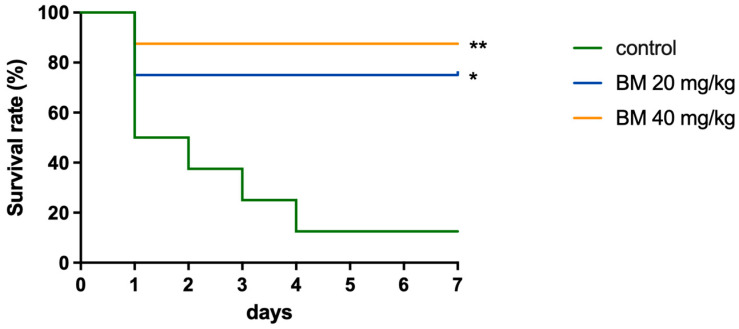
Baloxavir protects mice from lethal *S. aureus* sepsis. Survival curves of female ICR mice (*n* = 8 per group) challenged with *S. aureus* strain ATCC 33591. Mice received either vehicle control or the prodrug BM (20 or 40 mg/kg). Survival was monitored for 7 days post-infection. Statistical comparisons between treatment groups are indicated: * *p* < 0.05, ** *p* < 0.01, log-rank Mantel–Cox test).

*S. aureus* sepsis represents a clinically severe condition requiring immediate life-saving intervention; thus, demonstrating improved survival in a systemic infection model provides robust evidence of in vivo efficacy [20]. To evaluate in vivo efficacy, we employed a murine systemic infection model using MRSA ATCC 33591 (Figure 4). For in vivo studies, the prodrug BM was administered to optimize solubility and bioavailability.

In the vehicle control group, mice succumbed rapidly to infection, with only 12.5% (1/8) surviving at day 7. In contrast, mice treated with BM showed significantly improved survival. At 20 mg/kg, survival reached 75% (6/8; *p* = 0.019 vs. control), and at 40 mg/kg, survival was 87.5% (7/8; *p* = 0.007 vs. control). Notably, mortality in the treatment groups occurred exclusively within the first 24 h, suggesting that BM provided critical protection against the acute phase of lethal sepsis.

### 2.6. Baloxavir Suppresses De Novo Purine Biosynthesis and Activates VraSR-Mediated Cell Wall Stress

**Figure 5:**
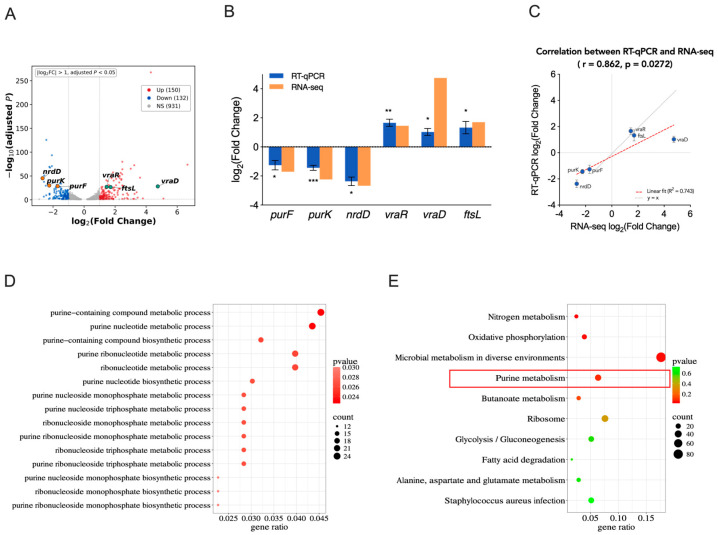
Transcriptional alterations in *S. aureus* induced by baloxavir treatment**.** (**A**) Volcano plot showing differential gene expression in *S. aureus* Newman treated with baloxavir (1/2 MIC for 3 h) vs. untreated controls analyzed by RNA-seq (*n* = 3 biological replicates). Genes with log_2_(fold change) > 1 or <−1 and adjusted *p* < 0.05 are highlighted. Red points indicate significantly upregulated genes; blue points indicate significantly downregulated genes. Key genes validated by RT-qPCR are highlighted in the volcano plot, including purine biosynthesis genes (*purF*, *purK*, *nrdD*; orange), cell wall stress genes (*vraR*, *vraD*; green), and cell division gene (*ftsL*; green). (**B**) RT-qPCR validation of six differentially expressed genes. Bars represent log_2_(fold change) in baloxavir-treated vs. untreated Newman strain. Data are mean ± SEM from three independent biological replicates. * *p* < 0.05, ** *p* < 0.01, *** *p* < 0.001 (Student’s *t*-test). (**C**) Correlation between RNA-seq and RT-qPCR fold changes for the six validated genes (Pearson correlation coefficient r = 0.862, *p* = 0.0272). (**D**) Selected GO terms related to purine metabolism and (**E**) Top 10 enriched KEGG pathways. Dot size indicates gene ratio; color intensity indicates adjusted *p*-value significance. The red frame highlights the purine metabolism pathway, which is highly relevant to the proposed antibacterial mechanism.

To elucidate the molecular mechanism underlying the antibacterial activity of baloxavir, we performed RNA-seq analysis comparing baloxavir-treated (1/2 MIC, 3 h) and untreated *S. aureus* Newman. Differential expression analysis identified 282 significantly altered genes (150 upregulated and 132 downregulated (|log_2_FC| > 1, *p*_adj < 0.05)) (Figure 5A, Table S4).

Gene Ontology (GO) enrichment analysis of differentially expressed genes revealed significant enrichment of purine metabolism-related processes, including purine-containing compound metabolic process and purine nucleotide metabolic process (Figure 5D). KEGG pathway analysis further confirmed these findings, with purine metabolism ranking among the top enriched pathways (Figure 5E).

Notably, genes encoding key enzymes in the early steps of purine nucleotide synthesis (*purF*, *purK*) and anaerobic nucleotide synthesis (*nrdD*) were significantly downregulated (log_2_FC = −1.70, −2.24 and −2.67, respectively). *PurF* initiates de novo purine biosynthesis by converting PRPP to phosphoribosylamine, while PurK functions in subsequent IMP synthesis steps. The concurrent downregulation of these enzymes would severely impair de novo purine nucleotide production. In contrast, genes related to the cell envelope stress response, including the two-component system regulators *vraR* and *vraD*, were significantly upregulated (log_2_FC = 1.45 and 4.78, respectively). This was accompanied by the induction of the cell division gene *ftsL* (log_2_FC = 1.70), suggesting activation of stress response pathways secondary to metabolic perturbation (Figure 5A,B).

To validate RNA-seq findings, we performed RT-qPCR analysis of six key genes representing distinct functional categories (Figure 5B). Consistent with transcriptomic data, significant downregulation was confirmed for *purF* (log_2_FC = −1.26 ± 0.32; *p* < 0.05), *purK* (log_2_FC = −1.44 ± 0.17; *p* < 0.001) and *nrdD* (log_2_FC = −2.37 ± 0.29; *p* < 0.05), and upregulations were confirmed for *vraR*, *vraD* and *ftsL* (log_2_FC = 1.66 ± 0.24, 1.03 ± 0.24, and 1.33 ± 0.43, respectively). RT-qPCR results showed strong concordance with RNA-seq data (Pearson r = 0.862, *p* = 0.0272) (Figure 5C).

## 3. Discussion

In this study, we report for the first time that baloxavir, an FDA-approved *influenza* antiviral, exhibits significant antibacterial activity against *S. aureus*, including MRSA and VISA strains. Our comprehensive evaluation demonstrated consistent in vitro activity across diverse strains (MIC_90_ = 4 μg/mL) and robust in vivo efficacy in a murine systemic infection model. Transcriptomic analysis revealed that baloxavir disrupts bacterial purine biosynthesis, representing a novel antibacterial mechanism distinct from its known antiviral activity. Importantly, baloxavir exhibited significant efficacy against intracellular *S. aureus*, addressing a critical therapeutic gap where conventional antibiotics often fail.

The selective activity of baloxavir against Gram-positive bacteria may be attributed to multiple factors. The outer membrane of Gram-negative bacteria constitutes a well-known permeability barrier that restricts the entry of hydrophobic compounds. However, the prodrug BM, which has different physicochemical properties, also failed to inhibit Gram-negative bacteria, suggesting that permeability is not the sole explanation. Additional contributing factors may include active efflux mechanisms and the absence or structural divergence of the molecular target in Gram-negative species. Future studies could explore the use of outer membrane permeabilizers (e.g., polymyxin B nonapeptide) to assess whether baloxavir can inhibit Gram-negative bacteria when the permeability barrier is bypassed, which would help distinguish between penetration-related and target-related mechanisms of intrinsic resistance.

Transcriptomic analysis demonstrated that baloxavir treatment results in significant downregulation of *purF* and *purK*, encoding key enzymes in the purine de novo biosynthesis pathway. This finding is noteworthy given recent validation of PurF as a high-value antibacterial target. Lamprecht et al. recently described JNJ-6640, a first-in-class PurF inhibitor with nanomolar activity against *Mycobacterium tuberculosis* and exceptional selectivity over human enzymes [21]. Bacteria depend more heavily on de novo purine synthesis compared to mammalian cells, which can utilize salvage pathways, providing a basis for selective toxicity [22]. For *S. aureus* specifically, de novo purine biosynthesis is essential for intracellular survival in purine-limited host environments [23] and is upregulated during biofilm formation and persistent infections [24]. Clinical MRSA strains causing persistent bacteremia exhibit elevated *purF* expression compared to resolving infections [24], and purR mutants—which derepress purine biosynthesis—are refractory to vancomycin treatment in endocarditis models [25]. These findings collectively highlight the essential role of purine biosynthesis in *S. aureus* survival within host cells and during persistent infections. Our transcriptomic data show that baloxavir treatment is associated with suppression of *purF* and *purK* expression, which, together with the effective eradication of intracellular *S. aureus* (Figure 3), raises the possibility that disruption of this pathway contributes to baloxavir’s antibacterial activity. However, supplementation with exogenous purine bases (adenine, guanine, and hypoxanthine) and nucleoside (guanosine) at concentrations of 0.2 and 1 mM did not alter the MIC of baloxavir. This is consistent with recent findings regarding JNJ-6640, even for this validated PurF inhibitor, exogenous adenine, guanine, and xanthine failed to rescue bactericidal activity in vitro, with only hypoxanthine showing partial rescue [21]. These results suggest that the downregulation of purine biosynthesis genes may represent a secondary transcriptional response to upstream perturbation rather than direct enzymatic inhibition.

**Figure 6:**
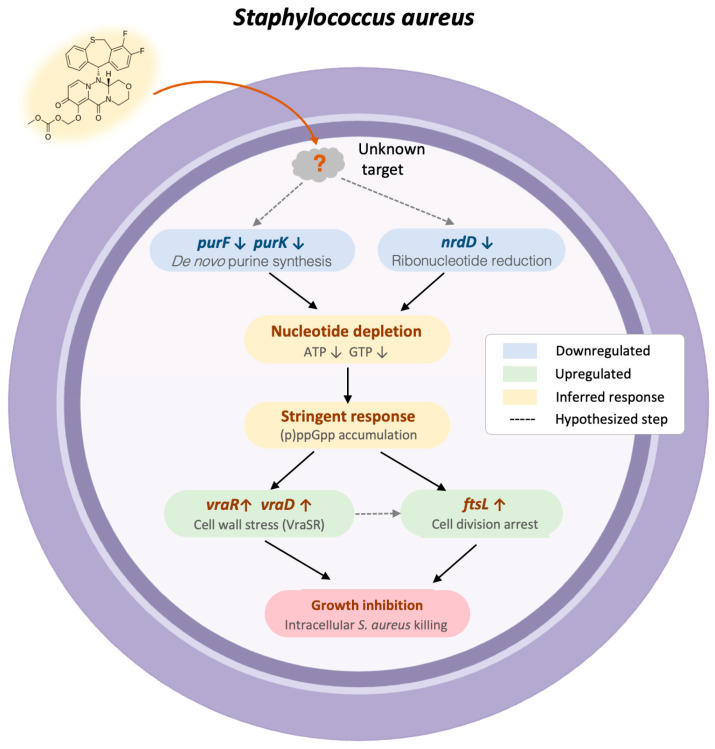
Proposed mechanistic model of baloxavir’s antibacterial activity in *S. aureus*. Baloxavir acts through an unidentified target to downregulate *purF*, *purK*, and *nrdD*, impairing de novo purine biosynthesis and causing nucleotide depletion. This triggers the stringent response via (p)ppGpp accumulation, activating VraSR-mediated cell wall stress (*vraR*, *vraD* upregulation) and cell division arrest (*ftsL* upregulation), ultimately leading to growth inhibition and intracellular killing. Dashed arrows indicate hypothesized steps.

Purine starvation leads to a depletion of ATP and GTP, which triggers the stringent response through (p)ppGpp accumulation [26]. Persister formation typically relies on (p)ppGpp-mediated dormancy, which confers antibiotic tolerance. However, even dormant bacteria require minimal nucleotide pools to survive. By blocking purine synthesis, baloxavir may disrupt this process—bacteria enter dormancy but cannot survive due to nucleotide depletion. This may explain our observation that baloxavir effectively cleared intracellular *S. aureus* (Figure 3), a niche where bacteria typically rely on stringent response-mediated dormancy to persist [27]. Importantly, (p)ppGpp accumulation is known to activate cell wall stress pathways through the VraSR two-component system, which is the primary sentinel for cell wall damage in *S. aureus* [28]. Consistent with this, we observed a robust upregulation of *vraR* and *vraD*, components of the VraSR regulon. Consequently, the upregulation of the cell division gene *ftsL* observed here is likely the consequence of VraSR-driven compensatory response. Collectively, these findings support a hypothetic mechanistic model: baloxavir inhibits purine de novo biosynthesis, causing nucleotide depletion that triggers the stringent response, activates cell wall stress pathways, and arrests cell division (Figure 6).

A particularly significant finding of this study is the potent intracellular bactericidal activity of baloxavir. It should be noted that the observed reduction in intracellular bacterial loads likely reflects a combination of direct drug-mediated growth inhibition and host innate immune responses, including ROS-mediated bacterial killing. *S. aureus* can persist for 3–4 days within macrophage phagosomes before escaping to the cytoplasm [29], and can survive up to 2 weeks within epithelial cells [30]. Most conventional antibiotics (β-lactams, vancomycin, aminoglycosides) are often ineffective against intracellular bacteria due to poor cellular penetration, reduced bacterial targets during metabolic dormancy, and active tolerance induction through stress responses [31], leading to recurrent infections and treatment failure [32]. Unlike conventional antibiotics that target processes active only during bacterial division, purine de novo biosynthesis is a fundamental pathway required for nucleotide homeostasis even during dormancy. If baloxavir indeed disrupts purine metabolism as our transcriptomic data suggest, it may remain effective against dormant persisters. Previous pharmacokinetic studies have demonstrated that baloxavir exhibits a large volume of distribution (1180 L, ~17 L/kg), far exceeding total body water and indicating extensive intracellular penetration that supports its efficacy against intracellular bacteria [33]. Interestingly, we observed superior intracellular killing efficacy in A549 epithelial cells compared to RAW264.7 macrophages. This difference may be attributed to the distinct metabolic states of intracellular bacteria between cell types. *S. aureus* within epithelial cells undergoes moderate multiplication following initial metabolic shutdown [30], whereas bacteria within macrophages may remain in a more dormant, tolerant state due to the harsher intravacuolar environment. If baloxavir’s mechanism requires active purine biosynthesis, the more metabolically active bacteria in epithelial cells would be more susceptible. Notably, the superior killing observed in A549 epithelial cells, which produce substantially less ROS than RAW264.7 macrophages, suggests that the direct antibacterial effect of baloxavir, rather than host ROS production, is the primary contributor to intracellular bacterial clearance.

The precise molecular target through which baloxavir inhibits bacterial purine biosynthesis requires further characterization. While its antiviral activity involves inhibition of *influenza* cap-dependent endonuclease through two-metal ion chelation, the bacterial target(s) remain to be identified since bacterial genomes lack homologous enzymes. However, enzymes utilizing the two-metal-ion catalytic mechanism are widespread in bacteria, including DNA and RNA polymerases, RNase P, and metallopeptidases. Given the strong chelating capacity of baloxavir acid for divalent metal ions (Mg^2+^ and Mn^2+^), it is plausible that baloxavir inhibits one or more of these essential metalloenzymes. Whether baloxavir directly inhibits PurF/PurK enzymatic activity or acts upstream to affect their transcription remains to be determined. The coordinated downregulation of multiple pathway genes observed in our study might suggest transcriptional repression rather than direct enzymatic inhibition of a single target. Future studies will focus on identifying the direct molecular target through several complementary approaches, including: (i) selection and whole-genome sequencing of spontaneous resistant mutants to identify target gene mutations; (ii) thermal proteome profiling to identify proteins whose thermal stability is altered upon baloxavir binding; and (iii) chemical proteomics using affinity probes derived from the baloxavir scaffold.

Beyond its established antiviral activity, the antibacterial properties of baloxavir identified in this study suggest a unique therapeutic opportunity for post-viral bacterial superinfection. *S. aureus* is a leading cause of secondary bacterial pneumonia following *influenza*, accounting for approximately 28% of bacterial co-infections in hospitalized patients and significantly increasing mortality risk [34]. *Influenza* infection upregulates host cell receptors and exposes fibronectin-binding sites on airway epithelium, creating a synergistic environment for staphylococcal invasion [35]. Notably, our finding that baloxavir effectively cleared intracellular *S. aureus* in A549 lung epithelial cells (Figure 3C,D)—the primary site of post-*influenza* bacterial colonization—suggests that a single agent could potentially address both the viral infection and the bacterial superinfection. Future studies will investigate baloxavir’s efficacy in *influenza*–*S. aureus* co-infection models to evaluate this dual therapeutic potential.

## 4. Materials and Methods

### 4.1. Bacterial Strains and Compounds

Standard strains were purchased from the American Type Culture Collection (ATCC, Manassas, VA, USA). Clinical isolates were obtained from the Collection Center of Pathogen Microorganism of Chinese Academy of Medical Sciences (CAMS-CCPM, Beijing, China). All strains were stored at −80 °C. For experiments, bacteria were recovered in 5 mL tryptic soy broth (TSB) and subcultured at 1:200 dilution in fresh TSB to logarithmic growth phase unless otherwise specified.

Baloxavir (catalog no. T14495, purity 99.87% by LC-MS) and the prodrug BM (catalog no. T6195, purity 99.89% by LC-MS) were purchased from TargetMol (Boston, MA, USA). Baloxavir was dissolved in dimethyl sulfoxide (DMSO) to prepare a stock solution at a concentration of 20 mg/mL, stored at −20 °C. Working solutions were prepared by serial dilution in culture medium. The final DMSO concentration in all assays did not exceed 1% (*v*/*v*), and equivalent DMSO concentrations were included in control groups for in vitro assays.

### 4.2. Antibacterial Spectrum Determination

The agar dilution method was performed according to Clinical and Laboratory Standards Institute (CLSI) guidelines [36]. Baloxavir was serially diluted two-fold from 128 to 0.03 μg/mL in 15 mL sterile cation-adjusted Mueller–Hinton (CAMH) agar. Both Gram-positive and Gram-negative bacterial strains were examined at a final inoculum of 10^4^ CFU per spot. A multipoint inoculator (Denley Instruments, London, UK) was used, and plates were incubated at 35 °C for 24 h. The MIC was defined as the lowest concentration inhibiting visible bacterial growth. Levofloxacin served as quality control.

### 4.3. Minimum Inhibitory Concentration (MIC) Determination

MICs against *S. aureus* strains were determined by broth microdilution as described previously [37]. Briefly, 100 μL of baloxavir serially diluted in CAMH broth (from 64 to 0.06 μg/mL) was added to 96-well plates. Log-phase bacterial suspension (10 μL, 5 × 10^6^ CFU/mL) was added to each well. Plates were incubated at 35 °C for 24 h. MIC values were determined as the lowest concentration showing no visible bacterial growth.

### 4.4. Time-Kill Assays

Time-kill studies were performed according to CLSI guidelines using five concentrations of baloxavir (1/4× to 4× MIC) and a growth control. Bacterial suspensions were prepared in CAMH broth at 10^6^ CFU/mL and incubated at 37 °C. At 0, 2, 4, 6, 8, and 24 h post-antibiotic exposure; then, 100 μL samples were serially diluted ten-fold in 0.9% saline, plated on tryptic soy agar (TSA) in triplicate, and incubated at 35 °C for 24 h. Bactericidal activity was defined as ≥3 log_10_ CFU/mL reduction from the initial inoculum.

### 4.5. Intracellular Antibacterial Activity

Intracellular MIC was determined following established protocols [38]. Both RAW264.7 macrophages and A549 epithelial cells (4 × 10^5^ cells/mL) were infected with *S. aureus* strains Newman or LAC at a multiplicity of infection (MOI) of 10. Gentamicin (50 μg/mL) was added to eliminate extracellular bacteria. Test antibiotics were introduced 24 h post-infection. Intracellular bacterial viability was assessed 24 h after antibiotic treatment by lysing cells with PBS containing 0.1% (*w*/*v*) Triton X-100 (Sigma, St. Louis, MO, USA). Lysates were serially diluted in PBS with 0.05% Tween-20 and plated on TSA for colony enumeration.

### 4.6. In Vivo Efficacy in Murine Systemic Infection Model

Female ICR mice (4–6 weeks old, 20–22 g) were obtained from SPF Biotechnology (Beijing, China). All animal experiments were performed according to national standards for laboratory animals in China (GB/T 35892-2018) [39] and approved by Animal Care & Welfare Committee of Institute of Medicinal Biotechnology, Chinese Academy of Medical Sciences & Peking Union Medical College (Approval No: IMB-20230220D_9_01, date of approval: 20 February 2023). Mice were randomly assigned to groups (*n* = 8) and challenged intraperitoneally with 0.5 mL of *S. aureus* ATCC 33591 (MRSA) at 10^6^ CFU/mouse. At 1 h post-infection, mice received intraperitoneal administration of BM (20 or 40 mg/kg) or vehicle. Survival was monitored for 7 days.

### 4.7. RNA Sequencing

*S. aureus* Newman was cultured to mid-logarithmic phase (OD_600_ ≈ 0.5) in TSB and exposed to baloxavir at 1/2 × MIC or PBS control for 3 h. Three biological replicates were prepared for each condition. Total RNA was extracted using RNeasy Mini Kit (QIAGEN, Hilden, Germany) following the manufacturer’s protocol. mRNA was purified from total RNA using probes to remove rRNA. Double-stranded cDNA was synthesized, followed by end repair, A-tailing, and adaptor ligation, and the cDNA libraries were sequenced on an Illumina Novaseq platform and 150 bp paired-end reads were generated (conducted by Novogene Co., Ltd., Beijing, China). Raw sequencing reads were quality-filtered using fastp with default parameters. Clean reads were aligned to the *S. aureus* NCTC8325 reference genome (GenBank: NC_007795.1) using Bowtie2. Gene expression quantification was performed using featureCounts. Differential expression analysis was conducted using DESeq2 with significance thresholds of |log_2_FC| > 1 and adjusted *p*-value < 0.05.

### 4.8. Quantitative Reverse Transcription PCR (RT-qPCR) Validation

The culture condition and extraction method of total RNA was the same as that of RNA sequencing. cDNA was synthesized from 0.5 μg total RNA using the HiScript II 1st Strand cDNA Synthesis Kit (Vazyme Biotech, Nanjing, China). Quantitative PCR was performed using SuperReal PreMix Plus (SYBR Green) (Tiangen, Beijing, China) on qTOWER^®3^ Real-Time PCR Systems (Analytik Jena, Jena, Germany). Primer sequences are listed in Supplementary Table S3. The housekeeping gene *gyrB* was used as the internal reference. Relative gene expression was calculated using the 2^−ΔΔCt^ method [40]. Three biological replicates with three technical replicates each were analyzed.

### 4.9. Statistical Analysis

All experiments were performed with at least three biological replicates. Quantitative data are presented as mean ± SEM. One-way ANOVA followed by Dunnett’s multiple comparisons test was used to compare treatment groups with the control. Survival curves were analyzed using the Kaplan–Meier method and compared using the log-rank (Mantel–Cox) test. A *p*-value < 0.05 was considered statistically significant. Significance levels are indicated as: * *p* < 0.05, ** *p* < 0.01, *** *p* < 0.001, **** *p* < 0.0001.

## 5. Conclusions

In conclusion, we have identified a novel antibacterial application for the approved *influenza* antiviral baloxavir. The compound demonstrates consistent activity against *S. aureus*, potent intracellular antibacterial effects, and promising in vivo efficacy. Mechanistic studies suggest disruption of purine biosynthesis as the underlying mechanism for its antibacterial activity. These findings support further development of baloxavir as a potential therapeutic option for staphylococcal infections, with particular promise for treating viral–bacterial co-infections.

## Data Availability

The RNA-seq data generated in this study have been deposited in the NCBI Sequence Read Archive (SRA) under accession number PRJNA1400452. All other data are included within the article and the Supplementary Files.

## Supplementary Materials

The following supporting information can be downloaded at: https://www.mdpi.com/article/10.3390/ijms27093880/s1.
