# Supplementary material for: Baloxavir Exhibits Antibacterial Activity Against Staphylococcus aureus by Inhibiting De Novo Purine Biosynthesis
Source: Int J Mol Sci. 2026 Apr 27;27(9):3880. doi: 10.3390/ijms27093880 (PMC13163285; doi:10.3390/ijms27093880)
Supplement: Supplementary file 1 [file ijms-27-03880-s001.zip › ijms-4240347-supplementary.pdf]

Supplementary materials

Table S1. Antibacterial spectrum of Baloxavir

**Species**	**Strains**	**Resistance phenotype**	**MIC (μg/mL)**
S. epidermidis	ATCC 12228	MSSE	4
	CCPM(A)-P-021901	MRSE	4
	CCPM(A)-P-021713	MSSE	4
S. aureus	ATCC 29213	MSSA	4
	CCPM(A)-P-012011	MRSA	4
	CCPM(A)-P-012012	MSSA	4
E. faecalis	ATCC 29212	VSE	4
	CCPM(A)-P-052002	VSE	2
	CCPM(A)-P-051806	VSE	4
E. faecium	ATCC 700221	VRE	2
	CCPM(A)-P-061506	VRE	2
	CCPM(A)-P-062002	VSE	<0.125
E. coli	ATCC 25922	Susceptible	>128
	ATCC 35218	Susceptible	>128
	CCPM(A)-P-072101	CRE	>128
K. pneumoniae	ATCC 700603	ESBL	>128
	ATCC BAA2146	CRE	>128
	CCPM(A)-P-082103	Susceptible	>128
P. aeruginosa	ATCC 27853	Susceptible	>128
	PAO1	Susceptible	>128
A. baumannii	ATCC 19606	Susceptible	>128
	CCPM(A)-P-101633	CRAB	>128

MSSE: methicillin-susceptible *Staphylococcus epidermidis*; MRSE: methicillin-resistant *Staphylococcus epidermidis*; MSSA: methicillin-susceptible *Staphylococcus aureus*; MRSA: methicillin-resistant *Staphylococcus aureus*; VRE: vancomycin-resistant *Enterococcus*; VSE: vancomycin-susceptible *Enterococcus*; CRE: carbapenem-resistant *Enterobacteriaceae*; ESBL: extended-spectrum β-lactamase; CRAB: carbapenem-resistant *Acinetobacter baumannii*.

Table S2. MICs of Baloxavir against *S. aureus* clinical isolates.

Strain	Source	Department	Resistance phenotype	MIC (μg/mL)
CCPM(A)-P-011778	blood	Neurology	MRSA	4
CCPM(A)-P-011697	blood	Neonatology	MRSA	2
CCPM(A)-P-0116168	blood	Nephrology	MRSA	4
CCPM(A)-P-0116173	blood	ICU	MRSA	4
CCPM(A)-P-0116207	blood	Pulmonology	MRSA	4
CCPM(A)-P-0116150	blood	Orthopedics	MSSA	4
CCPM(A)-P-011723	blood	Infectology	MSSA	4
CCPM(A)-P-011717	blood	Dermatology	MSSA	4
CCPM(A)-P-011711	blood	Infectology	MSSA	4
CCPM(A)-P-012026	blood	Infectology	MSSA	4
CCPM(A)-P-012110	secretions	Hepatobiliary	MRSA	2
CCPM(A)-P-011930	secretions	Dermatology	MRSA	2
CCPM(A)-P-011912	secretions	Dermatology	MRSA	2
CCPM(A)-P-012171	secretions	Endocrinology	MRSA	2
CCPM(A)-P-012161	secretions	Otolaryngology	MRSA	2
CCPM(A)-P-012012	secretions	Otolaryngology	MSSA	4
CCPM(A)-P-012013	secretions	Radiation Oncology	MSSA	4
CCPM(A)-P-012014	secretions	Otolaryngology	MSSA	4
CCPM(A)-P-012021	secretions	Dermatology	MSSA	4
CCPM(A)-P-012016	secretions	Dermatology	MSSA	4
CCPM(A)-P-011901	sputum	Geriatrics	MRSA	4
CCPM(A)-P-012133	sputum	Hematology	MRSA	4
CCPM(A)-P-012126	sputum	Neurosurgery	MRSA	4
CCPM(A)-P-012113	sputum	Trauma Surgery	MRSA	4
CCPM(A)-P-012106	sputum	Pulmonology	MRSA	4
CCPM(A)-P-011933	sputum	Pulmonology	MSSA	2
CCPM(A)-P-011932	sputum	Pulmonology	MSSA	4
CCPM(A)-P-011929	sputum	Pulmonology	MSSA	2
CCPM(A)-P-011922	sputum	Pulmonology	MSSA	2
CCPM(A)-P-011918	sputum	Pulmonology	MSSA	4

Table S3. Primers for qPCR

Gene	Primer (5'-3')	Tm (°C)	Product(bp)
nrdD	TTGCCACAGTTCGGGCATTT	61.11	144
	AGCACTAGAAGCGGTATGGG	59.25	
purF	AAAGGCGAGCGAGGATTAGG	59.89	102
	AGCATAACGGACGTGACCAA	59.68	
purK	ACCAAAGTTGTCCCGTTCATT	58.34	113
	TTGCCATCGTAGCCACCAAA	60.25	
ftsL	TGATACGCGAGGAAAGATTGC	58.8	148
	GTTCTCAAGGCTCATCCCCT	59.04	
vraR	TCGTCGCTTCTACACCATCC	59.55	80
	TGCCAAAGCCCATGAGTTGA	60.18	
vraD	TGTCGAAGGCAACAATGGAAG	59.12	134
	AACAAATGCTCTCGCTGCTG	59.48	
gyrB	TGATGCCGATGTGGATGGAG	59.89	113
	ACAACGGTGGCTGTGCAATA	60.54	

Table S4. Differentially expressed genes in *S. aureus* Newman following baloxavir treatment identified by RNA-seq analysis.

gene_ID	log_2FC	*p*_adj	gene_name	gene_description
**LO764_RS04230**	4.30449845	3.25E-268	*emp*	extracellular matrix protein-binding adhesin Emp
**LO764_RS08645**	-2.4324669	1.62E-126	*thrS*	threonine--tRNA ligase PF02824:TGS domain|PF03129:Anticodon binding domain|PF00587:tRNA synthetase class II core domain (G, H, P, S and T)|PF07973:Threonyl and Alanyl tRNA synthetase second additional domain
**LO764_RS00310**	-2.0741116	3.02E-94	*spa*	staphylococcal protein A PF04650:YSIRK type signal peptide|PF01476:LysM domain|PF00746:Gram positive anchor|PF02216:B domain
**LO764_RS07795**	2.46360317	1.36E-80	*LO764_RS07795*	alpha-glucosidase PF16657:Maltogenic Amylase, C-terminal domain|PF00128:Alpha amylase, catalytic domain
**LO764_RS03090**	6.66418535	1.85E-74	*vraX*	C1q-binding complement inhibitor VraX
**LO764_RS13805**	3.23426865	2.85E-73	*cwrA*	cell wall inhibition responsive protein CwrA
**LO764_RS02395**	-2.0742047	1.98E-71	*aaa*	autolysin/adhesin Aaa PF01476:LysM domain|PF05257:CHAP domain
**LO764_RS07435**	-2.2372921	5.52E-70	*ald*	alanine dehydrogenase PF01262:Alanine dehydrogenase/PNT, C-terminal domain|PF05222:Alanine dehydrogenase/PNT, N-terminal domain
**LO764_RS07430**	-2.1940553	2.42E-68	*tdcB*	bifunctional threonine ammonia-lyase/L-serine ammonia-lyase TdcB PF00291:Pyridoxal-phosphate dependent enzyme
**LO764_RS02575**	3.00688201	5.83E-66	*LO764_RS02575*	Veg family protein PF06257:Biofilm formation stimulator VEG
**LO764_RS13510**	2.46918913	9.85E-65	*gntK*	gluconokinase PF02782:FGGY family of carbohydrate kinases, C-terminal domain|PF00370:FGGY family of carbohydrate kinases, N-terminal domain
**LO764_RS03510**	1.86558422	3.49E-58	*LO764_RS03510*	hypothetical protein
**LO764_RS13070**	2.36219012	2.87E-57	*hlgA*	bi-component gamma-hemolysin HlgAB subunit A PF07968:Leukocidin/Hemolysin toxin family
**LO764_RS07800**	2.45611468	2.80E-54	*LO764_RS07800*	LacI family DNA-binding transcriptional regulator PF00356:Bacterial regulatory proteins, lacI family|PF13377:Periplasmic binding protein-like domain
**LO764_RS00285**	-2.5699816	6.17E-52	*LO764_RS00285*	oleate hydratase PF06100:MCRA family
**LO764_RS08885**	1.74120342	1.97E-51	*LO764_RS08885*	trypsin-like peptidase domain-containing protein PF13180:PDZ domain|PF13365:Trypsin-like peptidase domain
**LO764_RS08515**	2.27050226	4.02E-51	*LO764_RS08515*	DUF4930 family protein PF16284:Domain of unknown function (DUF4930)
**LO764_RS13505**	2.29703648	1.37E-50	*LO764_RS13505*	gluconate:H+ symporter PF02447:GntP family permease
**LO764_RS14570**	3.60693172	3.41E-47	*vraE*	peptide resistance ABC transporter permease subunit VraE PF02687:FtsX-like permease family

**LO764_RS12735**	1.76943432	4.27E-46	*LO764_RS12735*	zinc ribbon domain-containing protein
**LO764_RS03420**	2.29428657	4.86E-46	*LO764_RS03420*	M50 family metallopeptidase PF13398:Peptidase M50B-like
**LO764_RS14125**	-2.6755439	6.20E-46	*nrdD*	anaerobic ribonucleoside-triphosphate reductase PF13597:Anaerobic ribonucleoside-triphosphate reductase
**LO764_RS10560**	2.06840663	1.39E-44	*eap*	extracellular adherence protein Eap/Map PF03642:MAP domain
**LO764_RS13515**	2.41030272	3.81E-44	*LO764_RS13515*	GntR family transcriptional regulator PF00392:Bacterial regulatory proteins, gntR family|PF07729:FCD domain
**LO764_RS10270**	2.39594549	1.66E-42	*liaF*	cell wall-active antibiotics response protein LiaF PF09922:Cell wall-active antibiotics response 4TMS YvqF
**LO764_RS09875**	1.43542049	8.31E-42	*sgtB*	monofunctional peptidoglycan glycosyltransferase SgtB PF00912:Transglycosylase
**LO764_RS14395**	1.90904302	4.29E-41	*icaR*	ica operon transcriptional regulator IcaR PF00440:Bacterial regulatory proteins, tetR family
**LO764_RS09540**	1.51557259	6.21E-40	*LO764_RS09540*	peptidylprolyl isomerase PF00639:PPIC-type PPIASE domain
**LO764_RS14120**	-2.5824785	1.99E-38	*nrdG*	anaerobic ribonucleoside-triphosphate reductase activating protein PF13353:4Fe-4S single cluster domain|PF04055:Radical SAM superfamily
**LO764_RS03750**	2.26665037	4.21E-37	*LO764_RS03750*	fructose-specific PTS transporter subunit EIIC PF02378:Phosphotransferase system, EIIC|PF00359:Phosphoenolpyruvate-dependent sugar phosphotransferase system, EIIA 2|PF02302:PTS system, Lactose/Cellobiose specific IIB subunit
**LO764_RS03275**	-2.0245845	3.79E-36	*adhP*	alcohol dehydrogenase AdhP PF00107:Zinc-binding dehydrogenase|PF08240:Alcohol dehydrogenase GroES-like domain
**LO764_RS05100**	-1.566292	2.29E-35	*LO764_RS05100*	glucosaminidase domain-containing protein PF01832:Mannosyl-glycoprotein endo-beta-N-acetylglucosaminidase
**LO764_RS07420**	-1.4813647	1.43E-34	*norB*	multidrug efflux MFS transporter NorB PF07690:Major Facilitator Superfamily
**LO764_RS10265**	1.59233559	7.47E-32	*LO764_RS10265*	sensor histidine kinase PF02518:Histidine kinase-, DNA gyrase B-, and HSP90-like ATPase|PF07730:Histidine kinase
**LO764_RS05185**	-1.7676123	1.43E-31	*purL*	phosphoribosylformylglycinamidine synthase subunit PurL PF02769:AIR synthase related protein, C-terminal domain|PF00586:AIR synthase related protein, N-terminal domain
**LO764_RS00945**	-1.7589672	2.30E-31	*LO764_RS00945*	FAD-binding oxidoreductase PF00042:Globin|PF00970:Oxidoreductase FAD-binding domain
**LO764_RS14505**	2.06169187	2.80E-31	*LO764_RS14505*	SMP-30/gluconolactonase/LRE family protein PF08450:SMP-30/Gluconolaconase/LRE-like region
**LO764_RS12440**	2.46708934	6.39E-31	*LO764_RS12440*	hypothetical protein

**LO764_RS05165**	-2.2446166	8.73E-31	*purK*	5-(carboxyamino)imidazole ribonucleotide synthase PF02826:D-isomer specific 2-hydroxyacid dehydrogenase, NAD binding domain|PF02222:ATP-grasp domain
**LO764_RS04240**	-2.465438	2.66E-30	*LO764_RS04240*	thermonuclease family protein PF00565:Staphylococcal nuclease homologue
**LO764_RS00515**	-1.7552328	9.04E-30	*adhE*	bifunctional acetaldehyde-CoA/alcohol dehydrogenase PF00465:Iron-containing alcohol dehydrogenase|PF00171:Aldehyde dehydrogenase family
**LO764_RS05190**	-1.7064601	2.20E-29	*purF*	amidophosphoribosyltransferase PF00156:Phosphoribosyl transferase domain|PF13537:Glutamine amidotransferase domain
**LO764_RS11650**	2.51256929	2.20E-29	*LO764_RS11650*	PTS mannitol transporter subunit IICB PF02378:Phosphotransferase system, EIIC|PF02302:PTS system, Lactose/Cellobiose specific IIB subunit
**LO764_RS14565**	4.74828923	7.10E-29	*vraD*	peptide resistance ABC transporter ATP-binding subunit VraD PF00005:ABC transporter
**LO764_RS10260**	1.45359372	5.38E-28	*vraR*	two-component system response regulator VraR PF00196:Bacterial regulatory proteins, luxR family|PF00072:Response regulator receiver domain
**LO764_RS13795**	1.18397599	5.92E-28	*pruA*	L-glutamate gamma-semialdehyde dehydrogenase PF00171:Aldehyde dehydrogenase family
**LO764_RS05195**	-1.5409304	1.16E-27	*purM*	phosphoribosylformylglycinamidine cyclo-ligase PF02769:AIR synthase related protein, C-terminal domain|PF00586:AIR synthase related protein, N-terminal domain
**LO764_RS06075**	1.70472517	1.90E-27	*ftsL*	cell division protein FtsL PF04977:Septum formation initiator
**LO764_RS03570**	-1.6597727	1.96E-27	*LO764_RS03570*	LysM peptidoglycan-binding domain-containing protein PF05257:CHAP domain|PF01476:LysM domain
**LO764_RS00610**	1.12103812	4.82E-27	*LO764_RS00610*	aldehyde dehydrogenase family protein PF00171:Aldehyde dehydrogenase family
**LO764_RS01050**	-1.8290523	5.43E-27	*scdA*	iron-sulfur cluster repair di-iron protein ScdA PF04405:Domain of Unknown function (DUF542)|PF01814:Hemerythrin HHE cation binding domain
**LO764_RS01820**	1.93451761	7.13E-27	*LO764_RS01820*	BglG family transcription antiterminator PF05043:Mga helix-turn-helix domain|PF00359:Phosphoenolpyruvate-dependent sugar phosphotransferase system, EIIA 2|PF08279:HTH domain|PF00874:PRD domain
**LO764_RS04530**	1.42209992	1.05E-24	*LO764_RS04530*	Na+/H+ antiporter family protein PF03553:Na+/H+ antiporter family|PF13726:Na+-H+ antiporter family
**LO764_RS07480**	1.38017115	1.08E-24	*LO764_RS07480*	YppE family protein PF08807:Bacterial domain of unknown function (DUF1798)
**LO764_RS02430**	-1.4640436	1.99E-24	*gltB*	glutamate synthase large subunit PF00310:Glutamine amidotransferases class-II|PF04898:Glutamate synthase central domain|PF01493:GXGXG motif|PF01645:Conserved region in glutamate synthase
**LO764_RS00805**	2.29282685	2.59E-24	*LO764_RS00805*	M23 family metallopeptidase PF01551:Peptidase family M23

**LO764_RS01805**	1.86443105	2.81E-24	*LO764_RS01805*	PTS ascorbate transporter subunit IIC PF03611:PTS system sugar-specific permease component
**LO764_RS05085**	2.22568121	6.00E-24	*LO764_RS05085*	acyltransferase family protein PF01757:Acyltransferase family
**LO764_RS12560**	2.3022193	7.63E-24	*LO764_RS12560*	hypothetical protein
**LO764_RS13145**	1.13662753	1.17E-23	*LO764_RS13145*	membrane protein
**LO764_RS01185**	-1.814724	1.94E-23	*esxA*	WXG100 family type VII secretion effector EsxA PF06013:Proteins of 100 residues with WXG
**LO764_RS03010**	-1.9227598	2.23E-23	*sdrD*	MSCRAMM family adhesin SdrD PF04650:YSIRK type signal peptide|PF00746:Gram positive anchor|PF05738:Cna protein B-type domain|PF10425:C-terminus of bacterial fibrinogen-binding adhesin
**LO764_RS13965**	-1.4827609	5.01E-23	*LO764_RS13965*	fructosamine kinase family protein PF03881:Fructosamine kinase
**LO764_RS14050**	-1.0601521	2.04E-21	*LO764_RS14050*	fructose bisphosphate aldolase PF00274:Fructose-bisphosphate aldolase class-I
**LO764_RS08555**	1.20490926	2.29E-21	*LO764_RS08555*	AbrB family transcriptional regulator PF05145:Putative ammonia monooxygenase
**LO764_RS10565**	1.98314841	2.74E-21	*LO764_RS10565*	phospholipase
**LO764_RS00625**	-1.4993231	4.47E-21	*LO764_RS00625*	DUF4242 domain-containing protein PF14026:Protein of unknown function (DUF4242)
**LO764_RS11980**	-1.2092551	4.58E-21	*budA*	acetolactate decarboxylase PF03306:Alpha-acetolactate decarboxylase
**LO764_RS02020**	-1.034626	6.45E-21	*LO764_RS02020*	PepSY domain-containing protein PF03413:Peptidase propeptide and YPEB domain
**LO764_RS07045**	1.27182065	7.21E-21	*LO764_RS07045*	LCP family protein PF03816:Cell envelope-related transcriptional attenuator domain
**LO764_RS13525**	1.93661681	1.80E-20	*LO764_RS13525*	GTP pyrophosphokinase family protein PF04607:Region found in RelA / SpoT proteins
**LO764_RS12370**	-1.1734777	7.10E-20	*LO764_RS12370*	acyl-CoA/acyl-ACP dehydrogenase PF02770:Acyl-CoA dehydrogenase, middle domain|PF08028:Acyl-CoA dehydrogenase, C-terminal domain|PF02771:Acyl-CoA dehydrogenase, N-terminal domain
**LO764_RS05180**	-1.9149026	1.21E-19	*purQ*	phosphoribosylformylglycinamidine synthase I PF13507:CobB/CobQ-like glutamine amidotransferase domain
**LO764_RS05360**	1.59167828	1.33E-19	*LO764_RS05360*	DUF4064 domain-containing protein PF13273:Protein of unknown function (DUF4064)
**LO764_RS07425**	-1.3552456	1.33E-19	*LO764_RS07425*	amino acid permease PF13520:Amino acid permease
**LO764_RS08510**	2.18243092	1.34E-19	*LO764_RS08510*	hypothetical protein
**LO764_RS00940**	-1.6217498	2.93E-19	*LO764_RS00940*	hypothetical protein

**LO764_RS10335**	1.20953298	5.84E-19	*rlmD*	23S rRNA (uracil(1939)-C(5))-methyltransferase RlmD PF05958:tRNA (Uracil-5-)-methyltransferase
**LO764_RS11985**	-1.0733374	6.09E-19	*alsS*	acetolactate synthase AlsS PF00205:Thiamine pyrophosphate enzyme, central domain|PF02776:Thiamine pyrophosphate enzyme, N-terminal TPP binding domain|PF02775:Thiamine pyrophosphate enzyme, C-terminal TPP binding domain
**LO764_RS00630**	-1.7650569	1.86E-18	*LO764_RS00630*	ABC transporter ATP-binding protein PF00005:ABC transporter|PF07673:Protein of unknown function (DUF1602)
**LO764_RS01930**	1.27568044	2.81E-18	*LO764_RS01930*	acetyl-CoA C-acetyltransferase PF02803:Thiolase, C-terminal domain|PF00108:Thiolase, N-terminal domain
**LO764_RS03640**	1.54781074	3.08E-18	*LO764_RS03640*	hypothetical protein
**LO764_RS14500**	-1.1036851	4.37E-18	*LO764_RS14500*	YceI family protein PF04264:YceI-like domain
**LO764_RS05200**	-1.5114346	6.47E-18	*purN*	phosphoribosylglycinamide formyltransferase PF00551:Formyl transferase
**LO764_RS13870**	-1.2361428	1.96E-17	*isaA*	lytic transglycosylase IsaA PF01464:Transglycosylase SLT domain
**LO764_RS04590**	1.16633971	2.89E-17	*ygs*	S1 domain-containing post-transcriptional regulator Ygs PF00575:S1 RNA binding domain
**LO764_RS08995**	1.66669872	5.70E-17	*LO764_RS08995*	PepSY domain-containing protein PF03413:Peptidase propeptide and YPEB domain
**LO764_RS04235**	1.24346549	7.75E-17	*LO764_RS04235*	hypothetical protein
**LO764_RS04440**	3.3879758	1.72E-16	*LO764_RS04440*	YutD family protein PF06265:Protein of unknown function (DUF1027)
**LO764_RS12460**	-1.3447525	2.53E-16	*LO764_RS12460*	CHAP domain-containing protein PF05257:CHAP domain
**LO764_RS00920**	1.6276761	3.48E-16	*LO764_RS00920*	acyl CoA:acetate/3-ketoacid CoA transferase PF01144:Coenzyme A transferase
**LO764_RS02075**	-1.1538873	4.89E-16	*LO764_RS02075*	L-cystine transporter PF00375:Sodium:dicarboxylate symporter family
**LO764_RS06855**	1.42842566	6.93E-16	*LO764_RS06855*	hypothetical protein
**LO764_RS02100**	-1.912198	7.20E-16	*xpt*	xanthine phosphoribosyltransferase PF00156:Phosphoribosyl transferase domain
**LO764_RS12800**	-1.0649125	9.83E-16	*LO764_RS12800*	hypothetical protein
**LO764_RS13855**	-1.3730756	1.10E-15	*LO764_RS13855*	CHAP domain-containing protein PF05257:CHAP domain
**LO764_RS00800**	-1.5522339	1.16E-15	*LO764_RS00800*	FMN-dependent NADH-azoreductase PF02525:Flavodoxin-like fold
**LO764_RS13710**	2.15663096	1.83E-15	*cidA*	holin-like murein hydrolase modulator CidA PF03788:LrgA family
**LO764_RS12890**	1.33965115	3.15E-15	*LO764_RS12890*	Hsp20/alpha crystallin family protein PF00011:Hsp20/alpha crystallin family
**LO764_RS12515**	1.78217216	1.36E-14	*sdpB*	CPBP family intramembrane metalloprotease SdpB PF02517:CAAX protease self-immunity

**LO764_RS04640**	1.10899166	2.14E-14	*lepB*	signal peptidase I PF00717:Peptidase S24-like
**LO764_RS00425**	-1.5199168	2.47E-14	*LO764_RS00425*	superoxide dismutase PF00081:Iron/manganese superoxide dismutases, alpha-hairpin domain|PF02777:Iron/manganese superoxide dismutases, C-terminal domain
**LO764_RS10360**	2.77096332	2.84E-14	*gatC*	Asp-tRNA(Asn)/Glu-tRNA(Gln) amidotransferase subunit GatC PF02686:Glu-tRNAGln amidotransferase C subunit
**LO764_RS00935**	-1.793964	3.02E-14	*LO764_RS00935*	DUF488 domain-containing protein PF04343:Protein of unknown function, DUF488
**LO764_RS14530**	-1.0273866	4.62E-14	*bstA*	bacillithiol transferase BstA
**LO764_RS14030**	-1.0668529	6.08E-14	*LO764_RS14030*	L-lactate dehydrogenase PF00056:lactate/malate dehydrogenase, NAD binding domain|PF02866:lactate/malate dehydrogenase, alpha/beta C-terminal domain
**LO764_RS13830**	-1.2351106	9.15E-14	*LO764_RS13830*	NAD(P)/FAD-dependent oxidoreductase PF01593:Flavin containing amine oxidoreductase
**LO764_RS05175**	-2.2263389	1.05E-13	*purS*	phosphoribosylformylglycinamidine synthase subunit PurS PF02700:Phosphoribosylformylglycinamidine (FGAM) synthase
**LO764_RS04635**	1.24725504	1.80E-13	*lepB*	signal peptidase I PF00717:Peptidase S24-like
**LO764_RS05205**	-1.0327022	1.99E-13	*purH*	bifunctional phosphoribosylaminoimidazolecarboxamide formyltransferase/IMP cyclohydrolase PF02142:MGS-like domain|PF01808:AICARFT/IMPCHase bienzyme
**LO764_RS06870**	-1.1953835	2.18E-13	*thrC*	threonine synthase PF00291:Pyridoxal-phosphate dependent enzyme
**LO764_RS12235**	3.25416263	3.64E-13	*LO764_RS12235*	hypothetical protein
**LO764_RS00645**	-1.2594292	4.55E-13	*LO764_RS00645*	acyl-CoA/acyl-ACP dehydrogenase
**LO764_RS00635**	-1.5164624	7.55E-13	*LO764_RS00635*	ABC transporter substrate-binding protein PF13379:NMT1-like family
**LO764_RS04420**	1.30945866	1.20E-12	*LO764_RS04420*	DUF72 domain-containing protein PF01904:Protein of unknown function DUF72
**LO764_RS01345**	-2.2197466	1.43E-12	*LO764_RS01345*	5'-nucleotidase%2C lipoprotein e(P4) family PF03767:HAD superfamily, subfamily IIIB (Acid phosphatase)
**LO764_RS13760**	-1.2389672	1.98E-12	*clpL*	ATP-dependent Clp protease ATP-binding subunit ClpL PF10431:C-terminal, D2-small domain, of ClpB protein|PF07724:AAA domain (Cdc48 subfamily)|PF00004:ATPase family associated with various cellular activities (AAA)
**LO764_RS12860**	1.25498249	2.52E-12	*rsp*	AraC family transcriptional regulator Rsp PF12833:Helix-turn-helix domain
**LO764_RS04450**	1.60014249	5.13E-12	*LO764_RS04450*	DUF86 domain-containing protein PF01934:Protein of unknown function DUF86

**LO764_RS10430**	-1.0430682	7.27E-12	*LO764_RS10430*	nitric oxide synthase oxygenase PF02898:Nitric oxide synthase, oxygenase domain
**LO764_RS01810**	2.50864439	9.60E-12	*LO764_RS01810*	PTS sugar transporter subunit IIB PF02302:PTS system, Lactose/Cellobiose specific IIB subunit
**LO764_RS05160**	-2.2284817	1.30E-11	*purE*	5-(carboxyamino)imidazole ribonucleotide mutase PF00731:AIR carboxylase
**LO764_RS03700**	-1.0306355	2.12E-11	*LO764_RS03700*	DASS family sodium-coupled anion symporter PF00939:Sodium:sulfate symporter transmembrane region
**LO764_RS01815**	1.94582874	2.29E-11	*LO764_RS01815*	PTS sugar transporter subunit IIA PF00359:Phosphoenolpyruvate-dependent sugar phosphotransferase system, EIIA 2
**LO764_RS03745**	1.36651723	2.58E-11	*pfkB*	1-phosphofructokinase PF00294:pfkB family carbohydrate kinase
**LO764_RS11765**	2.17216779	3.13E-11	*sepA*	multidrug efflux transporter SepA PF17080:Multidrug Resistance efflux pump
**LO764_RS11175**	-1.0388026	4.24E-11	*LO764_RS11175*	SprT family protein PF10263:SprT-like family
**LO764_RS12435**	1.27481336	4.92E-11	*LO764_RS12435*	CHAP domain-containing protein PF05257:CHAP domain
**LO764_RS02105**	-1.4589005	5.83E-11	*LO764_RS02105*	purine permease PF00860:Permease family
**LO764_RS12255**	2.83812512	7.12E-11	*LO764_RS12255*	hypothetical protein
**LO764_RS14650**	1.43986982	9.06E-11	*rpmH*	50S ribosomal protein L34 PF00468:Ribosomal protein L34
**LO764_RS00735**	1.23686536	9.06E-11	*LO764_RS00735*	PTS transporter subunit EIIC PF00367:phosphotransferase system, EIIB|PF02378:Phosphotransferase system, EIIC
**LO764_RS10275**	1.50270644	9.07E-11	*LO764_RS10275*	hypothetical protein
**LO764_RS13615**	-1.2071314	9.15E-11	*LO764_RS13615*	D-lactate dehydrogenase PF02826:D-isomer specific 2-hydroxyacid dehydrogenase, NAD binding domain|PF00389:D-isomer specific 2-hydroxyacid dehydrogenase, catalytic domain
**LO764_RS13205**	-1.3450552	9.59E-11	*LO764_RS13205*	ABC transporter permease PF00528:Binding-protein-dependent transport system inner membrane component
**LO764_RS12590**	-1.1150888	1.72E-10	*LO764_RS12590*	SDR family oxidoreductase PF00106:short chain dehydrogenase
**LO764_RS13220**	-1.2765402	2.78E-10	*LO764_RS13220*	ABC transporter ATP-binding protein PF00005:ABC transporter|PF00571:CBS domain|PF07673:Protein of unknown function (DUF1602)
**LO764_RS00240**	-2.4392333	2.87E-10	*LO764_RS00240*	phosphatidylinositol-specific phospholipase C PF00388:Phosphatidylinositol-specific phospholipase C, X domain
**LO764_RS13725**	-1.446011	4.87E-10	*LO764_RS13725*	CHAP domain-containing protein PF05257:CHAP domain
**LO764_RS10320**	1.06577365	6.45E-10	*LO764_RS10320*	3'-5' exonuclease PF00929:Exonuclease

**LO764_RS13210**	-1.1948037	6.57E-10	*LO764_RS13210*	osmoprotectant ABC transporter substrate-binding protein PF04069:Substrate binding domain of ABC-type glycine betaine transport system
**LO764_RS04830**	1.22628479	1.11E-09	*LO764_RS04830*	hypothetical protein
**LO764_RS01080**	-1.8424018	1.66E-09	*LO764_RS01080*	glucose PTS transporter subunit IIA PF00358:phosphoenolpyruvate-dependent sugar phosphotransferase system, EIIA 1
**LO764_RS03765**	-1.0218655	2.12E-09	*LO764_RS03765*	aldo/keto reductase PF00248:Aldo/keto reductase family
**LO764_RS03410**	1.28208061	2.58E-09	*LO764_RS03410*	metal ABC transporter ATP-binding protein PF00005:ABC transporter
**LO764_RS00640**	-1.5272465	2.99E-09	*LO764_RS00640*	ABC transporter permease PF00528:Binding-protein-dependent transport system inner membrane component
**LO764_RS12870**	1.10460771	2.99E-09	*LO764_RS12870*	DUF4889 domain-containing protein PF16230:Domain of unknown function (DUF4889)
**LO764_RS04610**	-1.2587039	3.42E-09	*LO764_RS04610*	glycerophosphodiester phosphodiesterase PF03009:Glycerophosphoryl diester phosphodiesterase family
**LO764_RS02110**	-1.0721848	3.62E-09	*guaB*	IMP dehydrogenase PF00478:IMP dehydrogenase / GMP reductase domain|PF00571:CBS domain
**LO764_RS10570**	1.3045962	3.75E-09	*LO764_RS10570*	hypothetical protein
**LO764_RS11345**	1.4494846	4.07E-09	*LO764_RS11345*	single-stranded DNA-binding protein PF00436:Single-strand binding protein family
**LO764_RS06860**	-1.2493463	6.55E-09	*LO764_RS06860*	aspartate kinase PF00696:Amino acid kinase family
**LO764_RS13320**	-1.4517222	7.00E-09	*cntA*	staphylopine-dependent metal ABC transporter substrate-binding protein CntA PF00496:Bacterial extracellular solute-binding proteins, family 5 Middle
**LO764_RS06865**	-1.0511101	8.77E-09	*LO764_RS06865*	homoserine dehydrogenase PF00742:Homoserine dehydrogenase|PF03447:Homoserine dehydrogenase, NAD binding domain
**LO764_RS04200**	-1.6394037	9.10E-09	*LO764_RS04200*	DUF5067 domain-containing protein PF16729:Domain of unknown function (DUF5067)
**LO764_RS04245**	1.23482743	1.33E-08	*LO764_RS04245*	cold-shock protein PF00313:'Cold-shock' DNA-binding domain
**LO764_RS00825**	1.23945112	1.40E-08	*LO764_RS00825*	sugar ABC transporter permease PF00528:Binding-protein-dependent transport system inner membrane component
**LO764_RS11655**	1.1684256	1.48E-08	*LO764_RS11655*	BglG family transcription antiterminator PF00359:Phosphoenolpyruvate-dependent sugar phosphotransferase system, EIIA 2|PF02302:PTS system, Lactose/Cellobiose specific IIB subunit|PF00874:PRD domain|PF05043:Mga helix-turn-helix domain|PF08279:HTH domain
**LO764_RS02115**	-1.1365834	1.53E-08	*guaA*	glutamine-hydrolyzing GMP synthase PF00117:Glutamine amidotransferase class-I|PF00958:GMP synthase C terminal domain

**LO764_RS02195**	1.37620386	1.84E-08	*LO764_RS02195*	superantigen-like protein SSL9 PF09199:Staphylococcal superantigen-like OB-fold domain|PF02876:Staphylococcal/Streptococcal toxin, beta-grasp domain
**LO764_RS13215**	-1.3176705	2.19E-08	*LO764_RS13215*	ABC transporter permease PF00528:Binding-protein-dependent transport system inner membrane component
**LO764_RS00930**	-1.2044593	2.32E-08	*LO764_RS00930*	ABC transporter substrate-binding protein PF00496:Bacterial extracellular solute-binding proteins, family 5 Middle
**LO764_RS13470**	-1.1882628	3.00E-08	*LO764_RS13470*	E domain-containing protein PF04650:YSIRK type signal peptide|PF07501:G5 domain|PF17041:E domain
**LO764_RS13835**	-1.1550972	3.25E-08	*LO764_RS13835*	phytoene/squalene synthase family protein PF00494:Squalene/phytoene synthase
**LO764_RS01025**	1.00330849	4.23E-08	*LO764_RS01025*	CDP-glycerol--glycerophosphate glycerophosphotransferase PF04464:CDP-Glycerol:Poly(glycerophosphate) glycerophosphotransferase
**LO764_RS00740**	1.32113258	5.72E-08	*LO764_RS00740*	MurR/RpiR family transcriptional regulator PF01418:Helix-turn-helix domain, rpiR family|PF01380:SIS domain
**LO764_RS00960**	-1.2580826	6.12E-08	*LO764_RS00960*	PTS transporter subunit EIIC PF00367:phosphotransferase system, EIIB|PF02378:Phosphotransferase system, EIIC
**LO764_RS14080**	-1.4831522	6.14E-08	*betA*	choline dehydrogenase PF05199:GMC oxidoreductase|PF00732:GMC oxidoreductase
**LO764_RS06875**	-1.0833176	8.71E-08	*thrB*	homoserine kinase PF08544:GHMP kinases C terminal|PF00288:GHMP kinases N terminal domain
**LO764_RS12390**	-1.3516036	8.76E-08	*ureC*	urease subunit alpha PF00449:Urease alpha-subunit, N-terminal domain|PF01979:Amidohydrolase family
**LO764_RS01085**	-1.2218651	8.76E-08	*LO764_RS01085*	6-phospho-beta-glucosidase PF00232:Glycosyl hydrolase family 1
**LO764_RS02200**	1.11965114	9.86E-08	*LO764_RS02200*	superantigen-like protein SSL10 PF09199:Staphylococcal superantigen-like OB-fold domain|PF02876:Staphylococcal/Streptococcal toxin, beta-grasp domain
**LO764_RS00830**	1.15553462	1.03E-07	*LO764_RS00830*	Gfo/Idh/MocA family oxidoreductase PF01408:Oxidoreductase family, NAD-binding Rossmann fold
**LO764_RS04260**	1.61601121	1.14E-07	*LO764_RS04260*	hypothetical protein
**LO764_RS13565**	-1.7556092	1.74E-07	*LO764_RS13565*	ATP-binding cassette domain-containing protein PF00005:ABC transporter
**LO764_RS02170**	1.82805732	1.83E-07	*LO764_RS02170*	superantigen-like protein SSL4 PF09199:Staphylococcal superantigen-like OB-fold domain|PF02876:Staphylococcal/Streptococcal toxin, beta-grasp domain
**LO764_RS13690**	1.59451518	1.98E-07	*LO764_RS13690*	acyl-CoA thioesterase PF13279:Thioesterase-like superfamily
**LO764_RS14085**	-2.5933687	2.38E-07	*betB*	betaine-aldehyde dehydrogenase PF00171:Aldehyde dehydrogenase family

**LO764_RS02180**	1.67331841	2.58E-07	*LO764_RS02180*	superantigen-like protein SSL6 PF02876:Staphylococcal/Streptococcal toxin, beta-grasp domain|PF09199:Staphylococcal superantigen-like OB-fold domain
**LO764_RS07600**	1.30623489	3.71E-07	*LO764_RS07600*	heptaprenyl diphosphate synthase component 1 PF07307:Heptaprenyl diphosphate synthase (HEPPP synthase) subunit 1
**LO764_RS01180**	-1.930233	5.86E-07	*LO764_RS01180*	CHAP domain-containing protein PF05257:CHAP domain
**LO764_RS12400**	-1.1921129	6.47E-07	*LO764_RS12400*	urease accessory protein UreF PF01730:UreF
**LO764_RS10980**	1.02085344	7.24E-07	*mroQ*	CPBP family intramembrane metalloprotease MroQ PF02517:CAAX protease self-immunity
**LO764_RS05170**	-1.7514745	7.73E-07	*LO764_RS05170*	phosphoribosylaminoimidazolesuccinocarboxamide synthase PF01259:SAICAR synthetase
**LO764_RS05445**	2.02295477	1.28E-06	*LO764_RS05445*	YlbG family protein PF09902:Uncharacterized protein conserved in bacteria (DUF2129)
**LO764_RS03580**	1.02220801	1.41E-06	*LO764_RS03580*	AraC family transcriptional regulator PF12833:Helix-turn-helix domain
**LO764_RS00120**	1.04241777	1.77E-06	*LO764_RS00120*	MBL fold metallo-hydrolase PF00753:Metallo-beta-lactamase superfamily
**LO764_RS02380**	-1.1357386	2.16E-06	*LO764_RS02380*	methionine ABC transporter ATP-binding protein PF00005:ABC transporter|PF09383:NIL domain|PF07673:Protein of unknown function (DUF1602)
**LO764_RS10590**	1.19910132	2.44E-06	*LO764_RS10590*	SH3 domain-containing protein PF08460:Bacterial SH3 domain
**LO764_RS00590**	-1.0407214	2.75E-06	*cap8O*	type 8 capsular polysaccharide synthesis protein Cap8O PF00984:UDP-glucose/GDP-mannose dehydrogenase family, central domain|PF03720:UDP-glucose/GDP-mannose dehydrogenase family, UDP binding domain|PF03721:UDP-glucose/GDP-mannose dehydrogenase family, NAD binding domain
**LO764_RS06010**	-1.0576621	2.90E-06	*arcC*	carbamate kinase PF00696:Amino acid kinase family
**LO764_RS05945**	1.03922797	3.02E-06	*LO764_RS05945*	formyl peptide receptor-like 1 inhibitory protein PF16104:Formyl peptide receptor-like 1 inhibitory protein
**LO764_RS08140**	1.18691502	3.32E-06	*rpsU*	30S ribosomal protein S21 PF01165:Ribosomal protein S21
**LO764_RS11610**	1.44570937	3.57E-06	*LO764_RS11610*	hypothetical protein
**LO764_RS03740**	1.2453424	3.98E-06	*LO764_RS03740*	DeoR/GlpR family DNA-binding transcription regulator PF08220:DeoR-like helix-turn-helix domain|PF00455:DeoR C terminal sensor domain
**LO764_RS09370**	1.51266305	4.17E-06	*LO764_RS09370*	DUF4888 domain-containing protein PF16229:Domain of unknown function (DUF4888)
**LO764_RS07150**	1.15564568	5.90E-06	*LO764_RS07150*	hypothetical protein
**LO764_RS03900**	1.18742602	5.93E-06	*queF*	preQ(1) synthase PF14489:QueF-like protein

**LO764_RS02145**	-1.0289582	6.10E-06	*LO764_RS02145*	SDR family oxidoreductase PF05368:NmrA-like family
**LO764_RS12720**	1.15291671	6.45E-06	*LO764_RS12720*	TetR/AcrR family transcriptional regulator PF00440:Bacterial regulatory proteins, tetR family
**LO764_RS14040**	-1.2063009	6.80E-06	*LO764_RS14040*	aspartate aminotransferase family protein PF00202:Aminotransferase class-III
**LO764_RS03005**	-1.0274571	9.88E-06	*sdrC*	MSCRAMM family adhesin SdrC PF05738:Cna protein B-type domain|PF00746:Gram positive anchor|PF10425:C-terminus of bacterial fibrinogen-binding adhesin|PF04650:YSIRK type signal peptide
**LO764_RS05450**	1.21841027	1.82E-05	*LO764_RS05450*	hypothetical protein
**LO764_RS12930**	-1.9782926	2.00E-05	*narJ*	nitrate reductase molybdenum cofactor assembly chaperone
**LO764_RS06620**	1.45048372	2.75E-05	*LO764_RS06620*	hypothetical protein
**LO764_RS01190**	-1.0606155	3.13E-05	*esaA*	type VII secretion protein EsaA
**LO764_RS04225**	1.13382798	3.26E-05	*vwb*	von Willebrand factor binding protein Vwb PF08764:Staphylococcus aureus coagulase
**LO764_RS13315**	-1.3334353	3.34E-05	*LO764_RS13315*	ABC transporter permease PF00528:Binding-protein-dependent transport system inner membrane component
**LO764_RS09175**	3.45337134	3.34E-05	*LO764_RS09175*	hypothetical protein
**LO764_RS00965**	1.27011735	5.77E-05	*LO764_RS00965*	nucleoside hydrolase PF01156:Inosine-uridine preferring nucleoside hydrolase
**LO764_RS03715**	1.18964507	9.66E-05	*LO764_RS03715*	DUF1361 domain-containing protein PF07099:Protein of unknown function (DUF1361)
**LO764_RS04190**	-1.3549276	9.76E-05	*LO764_RS04190*	hypothetical protein
**LO764_RS11620**	1.02054228	9.99E-05	*LO764_RS11620*	SAP domain-containing protein PF02037:SAP domain
**LO764_RS10575**	1.15376133	0.00010048	*LO764_RS10575*	hypothetical protein
**LO764_RS05965**	1.0256269	0.00019246	*LO764_RS05965*	hypothetical protein
**LO764_RS13305**	-1.8614441	0.00019756	*LO764_RS13305*	ABC transporter ATP-binding protein PF00005:ABC transporter|PF07673:Protein of unknown function (DUF1602)
**LO764_RS14160**	-1.3257371	0.00019842	*LO764_RS14160*	hypothetical protein
**LO764_RS12410**	-1.0255701	0.0002552	*LO764_RS12410*	urease accessory protein UreD PF01774:UreD urease accessory protein
**LO764_RS05000**	2.76640688	0.00026941	*LO764_RS05000*	hypothetical protein
**LO764_RS02190**	1.12815619	0.00028926	*LO764_RS02190*	superantigen-like protein SSL8 PF02876:Staphylococcal/Streptococcal toxin, beta-grasp domain|PF09199:Staphylococcal superantigen-like OB-fold domain

**LO764_RS06005**	-1.0287176	0.00029074	*argF*	ornithine carbamoyltransferase PF00185:Aspartate/ornithine carbamoyltransferase, Asp/Orn binding domain|PF02729:Aspartate/ornithine carbamoyltransferase, carbamoyl-P binding domain
**LO764_RS00810**	1.74701574	0.00047907	*ugpC*	sn-glycerol-3-phosphate ABC transporter ATP-binding protein UgpC PF00005:ABC transporter|PF03459:TOBE domain
**LO764_RS12935**	-2.2084947	0.00049368	*narH*	nitrate reductase subunit beta PF13247:4Fe-4S dicluster domain|PF14711:Respiratory nitrate reductase beta C-terminal
**LO764_RS01210**	-1.1112883	0.00049724	*essC*	type VII secretion protein EssC PF12538:DNA transporter|PF01580:FtsK/SpoIIIE family
**LO764_RS10595**	-1.1436175	0.0008346	*sak*	staphylokinase PF02821:Staphylokinase/Streptokinase family
**LO764_RS02300**	1.22123948	0.00087351	*LO764_RS02300*	hypothetical protein
**LO764_RS05010**	1.80807697	0.00102702	*LO764_RS05010*	DoxX family protein PF07681:DoxX
**LO764_RS12895**	-1.8166388	0.00126733	*LO764_RS12895*	NarK/NasA family nitrate transporter PF07690:Major Facilitator Superfamily
**LO764_RS12955**	-1.6769735	0.0013287	*nirB*	nitrite reductase large subunit NirB PF03460:Nitrite/Sulfite reductase ferredoxin-like half domain|PF07992:Pyridine nucleotide-disulphide oxidoreductase|PF01077:Nitrite and sulphite reductase 4Fe-4S domain|PF04324:BFD-like [2Fe-2S] binding domain
**LO764_RS05290**	2.61334595	0.0013531	*LO764_RS05290*	hypothetical protein
**LO764_RS02305**	-1.3615667	0.00137658	*LO764_RS02305*	GTP-binding protein PF02492:CobW/HypB/UreG, nucleotide-binding domain|PF07683:Cobalamin synthesis protein cobW C-terminal domain
**LO764_RS01835**	1.05224086	0.00144289	*LO764_RS01835*	MepB family protein PF08877:MepB protein
**LO764_RS08520**	3.78907305	0.00154406	*LO764_RS08520*	hypothetical protein
**LO764_RS04995**	1.38337148	0.00166896	*LO764_RS04995*	hypothetical protein
**LO764_RS12940**	-1.9030469	0.00177933	*LO764_RS12940*	nitrate reductase subunit alpha PF01568:Molydopterin dinucleotide binding domain|PF14710:Respiratory nitrate reductase alpha N-terminal|PF00384:Molybdopterin oxidoreductase
**LO764_RS12950**	-1.6096826	0.00198506	*nirD*	nitrite reductase small subunit NirD PF13806:Rieske-like [2Fe-2S] domain
**LO764_RS13585**	-1.0030543	0.00212184	*LO764_RS13585*	alpha/beta hydrolase PF12695:Alpha/beta hydrolase family
**LO764_RS02140**	-1.3245087	0.00223565	*LO764_RS02140*	DUF1304 domain-containing protein PF06993:Protein of unknown function (DUF1304)
**LO764_RS12945**	-1.5805932	0.00262341	*cobA*	uroporphyrinogen-III C-methyltransferase PF00590:Tetrapyrrole (Corrin/Porphyrin) Methylases
**LO764_RS14585**	1.61023101	0.00272058	*LO764_RS14585*	hypothetical protein
**LO764_RS07025**	5.11433612	0.00298469	*LO764_RS07025*	hypothetical protein

**LO764_RS00370**	-1.1529823	0.00309979	*sbnH*	staphyloferrin B biosynthesis decarboxylase SbnH PF00278:Pyridoxal-dependent decarboxylase, C-terminal sheet domain|PF02784:Pyridoxal-dependent decarboxylase, pyridoxal binding domain
**LO764_RS00360**	-1.3332648	0.00337405	*sbnF*	3-(L-alanin-3-ylcarbamoyl)-2-[(2-aminoethylcarbamoyl)methyl]-2-hydroxypropanoate synthase SbnF PF06276:Ferric iron reductase FhuF-like transporter|PF04183:IucA / IucC family
**LO764_RS04195**	-1.5925291	0.00337405	*LO764_RS04195*	hypothetical protein
**LO764_RS14255**	-1.1313847	0.00398372	*aur*	zinc metalloproteinase aureolysin PF02868:Thermolysin metallopeptidase, alpha-helical domain|PF07504:Fungalysin/Thermolysin Propeptide Motif|PF01447:Thermolysin metallopeptidase, catalytic domain|PF03413:Peptidase propeptide and YPEB domain
**LO764_RS13325**	-1.3463488	0.00591782	*cntM*	staphylopine dehydrogenase CntM PF10100:Uncharacterized protein conserved in bacteria (DUF2338)
**LO764_RS13670**	1.8860692	0.00669425	*LO764_RS13670*	hypothetical protein
**LO764_RS13045**	1.39925258	0.00670589	*LO764_RS13045*	putative metal homeostasis protein
**LO764_RS05380**	1.20400598	0.00705465	*LO764_RS05380*	DUF5325 family protein
**LO764_RS08400**	1.06964301	0.00796856	*LO764_RS08400*	hypothetical protein
**LO764_RS14075**	1.06076369	0.00946691	*LO764_RS14075*	sterile alpha motif-like domain-containing protein PF06855:YozE SAM-like fold
**LO764_RS06785**	3.21992797	0.00998947	*LO764_RS06785*	minor capsid protein PF15542:Bacterial toxin 50|PF04233:Phage Mu protein F like protein
**LO764_RS05260**	1.23274007	0.0106161	*nrdH*	glutaredoxin-like protein NrdH PF00462:Glutaredoxin
**LO764_RS04735**	1.53992001	0.01069572	*LO764_RS04735*	DUF3899 domain-containing protein PF13038:Domain of unknown function (DUF3899)
**LO764_RS09155**	1.76405633	0.0109371	*LO764_RS09155*	competence protein ComK PF06338:ComK protein
**LO764_RS07710**	-1.3931191	0.01119325	*LO764_RS07710*	DUF1672 domain-containing protein PF07901:Protein of unknown function (DUF1672)
**LO764_RS02015**	1.60357889	0.01130954	*LO764_RS02015*	YxeA family protein PF06486:Protein of unknown function (DUF1093)
**LO764_RS13330**	-1.394995	0.01179083	*cntL*	D-histidine (S)-2-aminobutanoyltransferase CntL
**LO764_RS13910**	-1.3307991	0.01179083	*LO764_RS13910*	DUF2316 family protein PF10078:Uncharacterized protein conserved in bacteria (DUF2316)
**LO764_RS09150**	2.45849104	0.0119413	*sigS*	RNA polymerase sigma factor SigS PF04542:Sigma-70 region 2
**LO764_RS13945**	-1.3500092	0.01228929	*LO764_RS13945*	NAD(P)-binding domain-containing protein PF13738:Pyridine nucleotide-disulphide oxidoreductase
**LO764_RS08260**	3.16452635	0.01240525	*LO764_RS08260*	hypothetical protein

**LO764_RS00915**	1.35470832	0.01248749	*LO764_RS00915*	acyl--CoA ligase PF00501:AMP-binding enzyme|PF13193:AMP-binding enzyme C-terminal domain
**LO764_RS01220**	-1.9978792	0.01338693	*esxB*	WXG100 family type VII secretion effector EsxB PF06013:Proteins of 100 residues with WXG
**LO764_RS09675**	1.23471616	0.01383261	*LO764_RS09675*	tRNA-His
**LO764_RS01215**	-2.2659444	0.01569217	*esaC*	type VII secretion substrate EsaC
**LO764_RS14575**	2.82838363	0.01632589	*vraH*	peptide resistance ABC transporter activity modulator VraH
**LO764_RS11070**	1.42554487	0.01811581	*LO764_RS11070*	hypothetical protein
**LO764_RS00910**	1.86549138	0.02007099	*LO764_RS00910*	acyl-CoA dehydrogenase family protein PF02771:Acyl-CoA dehydrogenase, N-terminal domain|PF02770:Acyl-CoA dehydrogenase, middle domain|PF00441:Acyl-CoA dehydrogenase, C-terminal domain
**LO764_RS09885**	1.08016707	0.02056418	*LO764_RS09885*	hypothetical protein
**LO764_RS02160**	1.27154977	0.0236595	*LO764_RS02160*	superantigen-like protein SSL3 PF09199:Staphylococcal superantigen-like OB-fold domain|PF02876:Staphylococcal/Streptococcal toxin, beta-grasp domain
**LO764_RS14620**	-1.4590029	0.02462316	*LO764_RS14620*	DUF3147 family protein PF11345:Protein of unknown function (DUF3147)
**LO764_RS09670**	1.01297914	0.02482379	*LO764_RS09670*	tRNA-Gln
**LO764_RS00480**	-1.4040532	0.02592981	*LO764_RS00480*	phosphate/phosphite/phosphonate ABC transporter substrate-binding protein PF12974:ABC transporter, phosphonate, periplasmic substrate-binding protein
**LO764_RS13060**	1.89294333	0.02669059	*LO764_RS13060*	hypothetical protein
**LO764_RS14230**	-1.3171386	0.02671091	*arcD*	arginine-ornithine antiporter PF13520:Amino acid permease
**LO764_RS14250**	1.47085378	0.02828121	*LO764_RS14250*	arginine repressor PF01316:Arginine repressor, DNA binding domain|PF02863:Arginine repressor, C-terminal domain
**LO764_RS01090**	-1.1654013	0.03612437	*LO764_RS01090*	class I SAM-dependent methyltransferase PF08241:Methyltransferase domain
**LO764_RS06820**	1.06097148	0.03658588	*LO764_RS06820*	ABC transporter ATP-binding protein PF00005:ABC transporter
**LO764_RS03940**	-1.083936	0.03893782	*LO764_RS03940*	ABC transporter ATP-binding protein PF00005:ABC transporter
**LO764_RS14645**	1.21713043	0.04003506	*rnpA*	ribonuclease P protein component PF00825:Ribonuclease P
**LO764_RS14580**	1.23619711	0.04421712	*LO764_RS14580*	IS30 family transposase PF00665:Integrase core domain
